# Positron Emission Tomography (PET) Scan as a Diagnostic Tool for Giant Cell Arteritis

**DOI:** 10.7759/cureus.35835

**Published:** 2023-03-06

**Authors:** Akshaya Ramachandran, Drashti Antala, Prasun Pudasainee, Sreelakshmi Panginikkod

**Affiliations:** 1 Internal Medicine, Ascension Saint Francis Hospital, Evanston, USA; 2 Rheumatology Immunology and Allergy, Tufts University School of Medicine, Boston, USA

**Keywords:** temporal artery biopsy, steroids, skip lesions, fdg pet-ct, giant cell arteritis (gca)

## Abstract

Giant cell arteritis (GCA) is an inflammatory vasculitis that typically affects the elderly, preferentially involving large and medium-sized arteries and can potentially cause irreversible loss of vision. Early diagnosis and treatment are necessary to prevent this dreaded complication. Temporal artery biopsy has been the gold standard test in diagnosing GCA, however, false negative results due to presence of skip lesions, restricted inflammation, and early initiation of steroids have limited its diagnostic significance. We report a case of a 67-year-old female with headache, blurry vision, posterior scalp tenderness, feeble left temporal artery pulse on a physical exam with normal inflammatory markers. Temporal artery biopsy showed disruption and reduplication of internal elastic lamina without any evidence of giant cells or inflammatory cells. Owing to high clinical suspicion, fluorodeoxyglucose (FDG)-positron emission tomography (PET)/computed tomography (CT) was further done which revealed mildly increased uptake in the thoracic aorta, consistent with a diagnosis of large vessel vasculitis.

## Introduction

Giant cell arteritis (GCA) is a large and medium vessel vasculitis that predominantly affects the thoracic aorta and its branches. In the 1930s, Horton and colleagues first recognized GCA as a disease and described the characteristic histologic appearance of granulomatous inflammation of the temporal vessel [1]. The incidence rates of GCA increase progressively after 50 years of age.

The diagnosis of GCA is straightforward in patients with typical features of temporal artery involvement like headache, scalp tenderness, jaw claudication, and vision changes. However, GCA is characterized by a wide range of clinical manifestations and can present with atypical features, resulting in a challenging diagnosis. Extracranial involvement comprising the abdominal and thoracic branches of the aorta is seen in about 10-15% of the cases and can be associated with or without its cranial counterpart [2]. The primary purpose of making a prompt diagnosis of GCA is to prevent the most feared complication of irreversible visual loss due to ischemic neuropathy. While temporal artery biopsy (TAB) has been the gold standard in identifying GCA, false negative results due to the presence of skip lesions, restricted inflammation and the early necessity to initiate steroids have limited its diagnostic significance. According to the American College of Rheumatology (ACR) guidelines for the management of GCA, in patients with suspected GCA and a negative TAB result, diagnosis with noninvasive vascular imaging such as magnetic resonance imaging (MRI) or computed tomography (CT) angiography, ultrasonography, and 18F-fluorodeoxyglucose positron emission tomography (FDG-PET) has been recommended as a tool to provide additional evidence of the disease [3]. We present a case of GCA in a 67-year-old female diagnosed with an FDG-PET/CT exhibiting increasing uptake in the thoracic aorta. The role of FDG-PET/CT in the diagnosis of large vessel vasculitis has been investigated through multiple studies with the objective of minimizing invasiveness as well as timely intervention in the setting of high clinical suspicion [4-10]. The purpose of this case is to highlight the value of FDG-PET/CT in diagnosing GCA and its utility, particularly in the context of negative TAB.

## Case presentation

**Figure 1:**
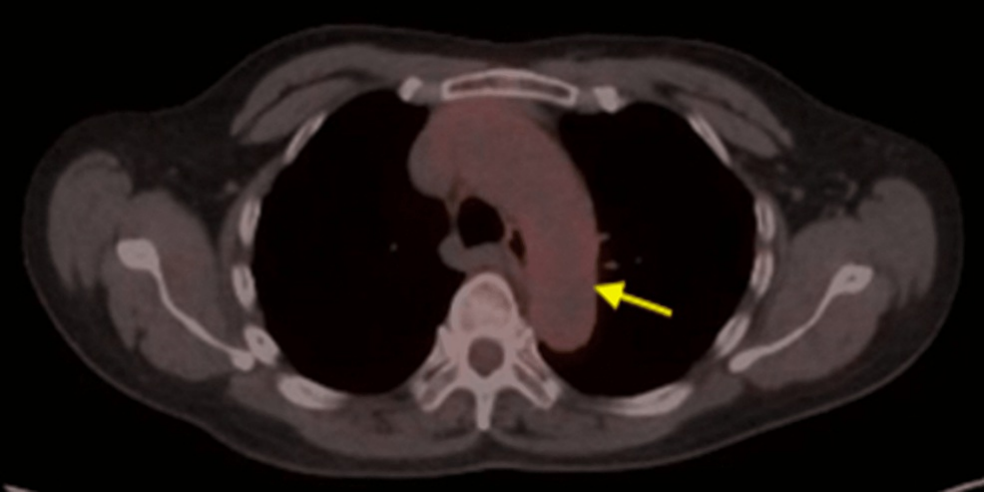
FDG-PET/CT of the chest Yellow arrow: Mild FDG uptake along the thoracic aorta FDG: fluorodeoxyglucose; PET: positron emission tomography; CT: computed tomography

A 67-year-old Asian female with a past medical history of gastroesophageal reflux disease (GERD), osteoarthritis, monoclonal gammopathy of undetermined significance (MGUS) presented with headache, fever, night sweats, fatigue, chest pain, generalized body pain, blurry vision, posterior scalp pain, neck pain, and 20-pound weight loss for six months. No h/o cough, sore throat, or difficulty in breathing. No h/o abdominal pain or swelling. No h/o joint swelling, redness, or warmth. No h/o blood in stool or urine. Physical examination was remarkable for posterior scalp tenderness, feeble left temporal artery pulse, and generalized tenderness over the bones. Owing to concern for large vessel vasculitis, she was immediately evaluated by ophthalmology and was found to have a normal fundus. With a presumptive diagnosis of polymyalgia rheumatica (PMR), she was put on a trial of low-dose prednisone and subsequently underwent a TAB. Erythrocyte sedimentation rate (ESR), C-reactive protein (CRP), and IgG 4 levels were normal, and antinuclear antibody (ANA) and antineutrophil cytoplasmic antibodies (ANCA) were negative. CT angiogram of the chest did not reveal any evidence of large vessel vasculitis. Her serum protein electrophoresis (SPEP) revealed increased gamma globulin fraction, serum immunofixation, and the skeletal survey was normal. TAB revealed disruption and reduplication of internal elastic lamina without any evidence of giant cells or inflammatory cells. Although TAB of GCA patients can show structural degenerative changes in the absence of inflammation, such as a media-intimal scar, intimal hyperplasia, fragmentation of internal elastic lamina, calcifications, or fibrosis of the adventitia, these changes are not specific to GCA because they are also found in unaffected older adults. She continued to experience generalized body pain and night sweats and was found to have multiple myofascial tender points on the exam. Considering the extensive workup that failed to identify the reason behind her symptoms, a working diagnosis of fibromyalgia was made and she was started on a trial of pregabalin with minimal improvement. As TABs can be negative due to the prevalence of skip lesions in GCA, further diagnostic studies were sought. The patient underwent an FDG-PET/CT scan which revealed mildly increased uptake in the thoracic aorta (Figure 1), consistent with a diagnosis of large vessel vasculitis, there was no evidence of FDG avid skeletal lesions or lymphadenopathy. She was started on Solumedrol 48 mg daily followed by the addition of tocilizumab.

## Discussion

GCA is an immune-mediated inflammatory disease of medium and large blood vessels resulting in a multitude of vascular complications. Although GCA tends to have a higher inclination towards the temporal artery, it can also involve the aorta, subclavian, iliac, ophthalmic, occipital, and vertebral arteries. It is a disease most commonly seen in the elderly population greater than 50 years of age, peaking at 70 years along with a female preponderance [2,11]. The classical symptoms that occur mostly due to the involvement of the cranial aortic branches include headache, jaw claudication, scalp tenderness, systemic symptoms such as fever, fatigue, arthralgia, and myalgia, and the most dreaded complication of vision loss [11].

The pathogenesis of GCA is a complex interplay between CD4T cell subtypes resulting in widespread inflammation and vascular occlusion. Endothelial injury due to trauma, infection, drugs, or autoantigens activate toll-like receptors (TLR) and trigger adventitial dendritic cells to release chemokines (CCL18-21) which further engage and recruit CD4 T-helper cells and macrophages into the arterial wall. The interleukins (IL-6, IL-8, IL-17, IL-12) secreted by the dendritic cells act on the CD4 cells that divide into Th17 and Th1 cells which release interferon-gamma (IFN-y) under the influence of which monocytes are transformed into multinucleated giant cells or macrophages. The activated macrophages then release reactive oxygen species (ROS), matrix metalloproteinase (MMP), and platelet-derived growth factor (PDGF) which destroy the vascular media and cause intimal hyperplasia leading to luminal stenosis [2,11].

The diagnosis of GCA can pose a challenge due to the indefinite character of its symptoms, especially in patients where the superficial cranial arteries might not be involved. TAB is considered to be the gold standard in diagnosing GCA with a specificity of 90-95%. However, there are multiple reasons contributing to false negative results. Biopsy samples must be obtained with an adequate size of at least 1 cm with three specimens at different levels to ensure a proper diagnosis. The presence of skip lesions, undersized sampling, circumscribed inflammation that can be missed out in one of the specimens, sparing of temporal arteries in large vessel involvement, and early initiation of steroids are factors that limit the diagnostic capacity of TAB in addition to its expensive and invasive nature [12]. In order to tackle these limitations, diagnostic modalities such as Doppler ultrasound, MRI, and PET scans have been explored to hasten diagnosis thereby leading to timely management and preventing major complications.

The imaging modality of interest in our case is the FDG-PET/CT scan which is a functional imaging technique more frequently used in the diagnosis of oncological conditions. Inflammatory cells have an elevated glycolytic activity resulting in an increased uptake of glucose (or glucose analogs such as FDG) thereby detecting abnormal metabolic activity in cancerous and inflammatory cells. A meta-analysis done by Soussan et al. showed that the FDG-PET scan was found to have a high sensitivity (pooled SN 90% with 95% CI of 79-93%) and specificity (pooled SP 98% with 95% CI of 94-99%) in diagnosing large vessel inflammation in GCA cases compared to controls [13]. A retrospective case-control study found that FDG-PET scan had a 79% sensitivity and a 92% specificity for the detection of cranial GCA [7,14]. It serves as a beneficial tool in cases where the diagnosis remains a challenge despite extensive evaluation and in cases where malignancy is suspected, much similar to our patient [15]. The European League Against Rheumatism (EULAR) guidelines on utilizing imaging modalities for the diagnosis and monitoring of large vessel vasculitis have provided a comprehensive assessment including the advantages and disadvantages of various imaging tools, which can be applied to practice depending upon each patient’s clinical scenario and pretest probability. According to these guidelines, the major advantage of PET/CT is its ability to detect wall luminal and thickness changes as well as other coexisting pathologies such as infections and tumors.

GCA can be frequently seen coexisting with PMR, given that they both belong to the same spectrum [5]. Therefore, when suspecting PMR in patients, clinicians should be vigilant for the presence of concurrent GCA. About 50% of patients with GCA have PMR and about 20% of PMR cases are associated with GCA [4,7]. According to a meta-analysis of three studies performed, the diagnostic accuracy of FDG-PET/CT in PMR was obtained with a pooled sensitivity and specificity of 85% and 80%, respectively [4]. When there is an uncertainty pertaining to the same, large-vessel imaging should be considered.

Nonetheless, FDG-PET/CT contains its own set of limitations. Interpretation of results is a challenge due to the lack of internationally recognized quantitative diagnostic values and is rather a qualitative measure based on the glucose uptake pattern [5,9,14,16,17]. While various studies have attempted to compare the diagnostic value of visual grading systems with semi-quantitative tools (SUV max), there is not sufficient data regarding the same in the literature and further studies need to be employed [5,9,13,14,16-18]. Furthermore, vascular uptake can increase with age and atherosclerosis, both of which can overestimate the rate of vascular inflammation in old-age patients [13,19,20]. Treatment with steroids can decrease the metabolic activity at the area of inflammation and thus its impact on PET scan yield needs to be explored further. In a retrospective study on cases with suspected GCA but negative TAB, corticosteroid therapy was not found to have any significant impact on the diagnostic performance of FDG-PET/CT, however, a trend of lower sensitivity was seen in those receiving steroids for more than three days [21]. As per a study done by de Boysson et al., long-term persistent vascular uptake on FDG-PET/CT scan was found in >80% of GCA patients who had a clinically and biologically controlled disease, and three of 25 cases studied had shown worsening of vascular intake even in absence of relapse [22]. Therefore the role of FDG-PET to monitor disease activity and predict relapses needs to be studied further.

## Conclusions

FDG-PET/CT can serve as a beneficial tool in the diagnosis of large vessel vasculitis, in patients who have undergone extensive evaluation including TAB. However, due to age and treatment-related confounding factors, there lacks a general consensus regarding its interpretation.
